# Geometric Parameters Estimation and Calibration in Cone-Beam Micro-CT

**DOI:** 10.3390/s150922811

**Published:** 2015-09-09

**Authors:** Jintao Zhao, Xiaodong Hu, Jing Zou, Xiaotang Hu

**Affiliations:** State Key Laboratory of Precision Measuring Technology and Instrument, Tianjin University, Tianjin 300072, China; E-Mails: lhjzjt@tju.edu.cn (J.T.Z.); xdhu@tju.edu.cn (X.D.H.); xthu@tju.edu.cn (X.T.H.)

**Keywords:** computed tomography, misalignment, geometric parameters estimation, calibration

## Abstract

The quality of Computed Tomography (CT) images crucially depends on the precise knowledge of the scanner geometry. Therefore, it is necessary to estimate and calibrate the misalignments before image acquisition. In this paper, a Two-Piece-Ball (TPB) phantom is used to estimate a set of parameters that describe the geometry of a cone-beam CT system. Only multiple projections of the TPB phantom at one position are required, which can avoid the rotation errors when acquiring multi-angle projections. Also, a corresponding algorithm is derived. The performance of the method is evaluated through simulation and experimental data. The results demonstrated that the proposed method is valid and easy to implement. Furthermore, the experimental results from the Micro-CT system demonstrate the ability to reduce artifacts and improve image quality through geometric parameter calibration.

## 1. Introduction

Micro-Computed Tomography (Micro-CT) is widely used for medical diagnosis and non-destructive testing (NDT) since the 1970s [1,2]. In three-dimensional (3D) Micro-CT imaging, the quality of the reconstructed images depends highly on the precision of the geometric parameters of the system. Image qualities would be severely degraded by misalignment parameters, such as more obvious artifacts, lower resolution, and more blurred images. Therefore, high-precision methods for estimating CT system parameters are necessary.

Since the 1980s, different research groups have proposed various kinds of methods to estimate geometric parameters for fan-beam CT or cone-beam CT [3,4,5,6]. In terms of adopted data acquisition methods, they can be categorized as multi-angles projection and single-angle projection [7,8,9,10]. The methods based on multi-angles of projection estimate the geometry parameters by using phantom projections at different angles. For example, Noo *et al.* [10] gave out a totally analytic method for estimating six parameters under the assumption that the detector and the rotation axis were parallel by making the precision rotation stage turning 360°. Lorenz von Smekal *et al.* [11] presented a high-precision method to determine the complete misalignment parameters based on Fourier analysis through multiple projection images. Kai Yang *et al.* [12] proposed an analytic method for deriving five system parameters with assuming that the detector does not have serve out-of-plane rotation (<2°) by using multiple projection images and angle information.

All the methods mentioned above could estimate some or all the geometric parameters by using analytic or iteration equations in the range of acceptable accuracy. However, a potential weakness is that they rely on multi-projections at different angles. This means that the precision of calibration would be affected by the errors of the rotation, such as radial or axial errors whose effect would be obvious in micro-CT, while it could be ignored in industrial CT. One way to eliminate the effects of rotation axis is to estimate the geometry parameters by acquiring the projection of corresponding phantom at only one angle.

Young bin cho *et al.* [13] developed a general analytic algorithm for estimating all the geometric parameters in cone-beam systems by using a corresponding phantom which consisted of 24 steel ball bearings in a known geometry. However, the phantom demands to distribute the 24 steel balls on two circles perfectly. If not, the precision of estimating geometric parameters would be reduced. Y. Sun *et al*. [14] proposed a method to estimate all parameters of the scanner geometry for calibration with a four-point phantom. However, in Sun’s method, the uncertainty of the scanner parameters was greatly influenced by the accuracy of the balls. In addition, only simulation results were shown.

In this paper, we proposed an “iterative” method which belongs to the latter methods. To do the estimation, we designed a TPB (Two-Piece-Ball represents two flat planes with nine ruby balls in each plane) phantom. The advantage of the proposed method is that only a single projection of the phantom at one angle every time is required and corresponding algorithm is derived. Furthermore, in previous literatures, authors always consider seven parameters to describe the geometric relationship. Here, we solve nine, increasing two offsets of the X-ray source spot. Normally, these two errors are always converted to the errors of the detector. However, if the phantom is in a plane or taking the focal spot drifts into consideration, as shown in the proposed method, this conversion would be improper. Additionally, we designed an adjusting mechanism to adjust the location of the detector. The angle of the detector would be adjusted every time basing on the calibration parameters until it approaches to zero or matches to our needs. Normally, three times is enough to adjust the system to a relatively ideal condition.

## 2. Geometry Definition and System Parameters

Generally, a micro-CT consists of an X-ray source, an X-ray planar detector, and a precision rotation stage using for placing samples which is located between the source and detector. In Micro-CT systems, the sample is rotated in the X-ray beam, while the X-ray source and the detector remain stationary.

**Figure 1:**
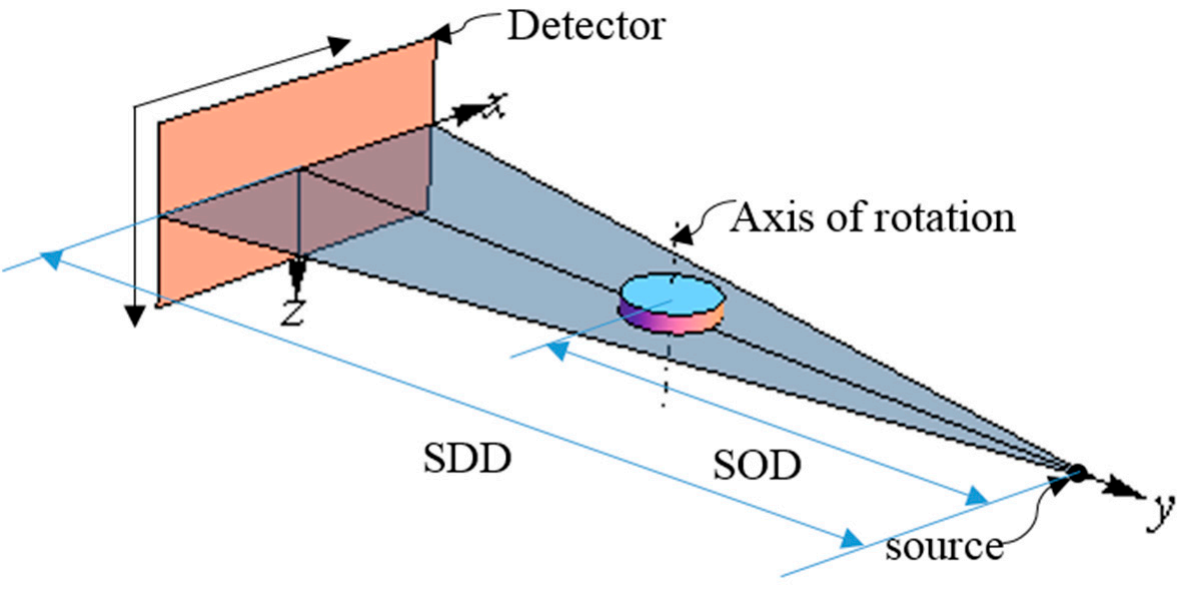
The ideal geometry of a micro-CT system.

In an ideal Micro-CT system, perfect alignment must be satisfied. It can be summarized as follows: firstly, the X-ray focal spot, the axis of the rotation, and the center of the detector should be in a plane; secondly, the straight line, which passes the focal spot and the detector center, should be vertical with the axis of rotation and the detector; thirdly, the axis of the rotation should be parallel to the row of the detector, as shown in Figure 1.

To describe the ideal case, we define a right-handed system of Cartesian coordinates X, Y, and Z. As shown in Figure 1, plane P denotes the detector planar, the center of the detector planar is the coordinate system origin, the X axis points to the ascending column direction, the Z axis points to the ascending row direction, and the Y axis is the normal of the detector and coincident with the central X-ray. The axis of the rotation intersects with the central X-ray.

It is well known that no scanner system can be free from misalignments. These misalignments can be concluded into three parts, that is, misalignments of the detector, the precision rotation stage, and the source. The misalignments of the rotation can be converted to that of the detector as analyzed in reference [8].

Therefore, we conclude all the misalignments or parameters as follows:

(1) μ_1_ and v_1_ are detector offsets along X and Z axis;
(2) α is the angle of detector tilt around X axis;
(3) β is the angle of detector tilt around Z axis;
(4) γ is the angle of detector tilt around Y axis;
(5) μ_2_ and v_2_ are X-ray source offsets along X and Z axis;
(6) SOD is the distance between X-ray source and rotation axis, and its estimated value is written as f;
(7) SDD is the perpendicular distance from X-ray source to detector.

That is to say there are a total of nine parameters to describe the scanner system. The purpose of our method is to get the nine geometric parameters according to the projection of a phantom collected at one angle and adjust the location of the detector.

**Figure 2:**
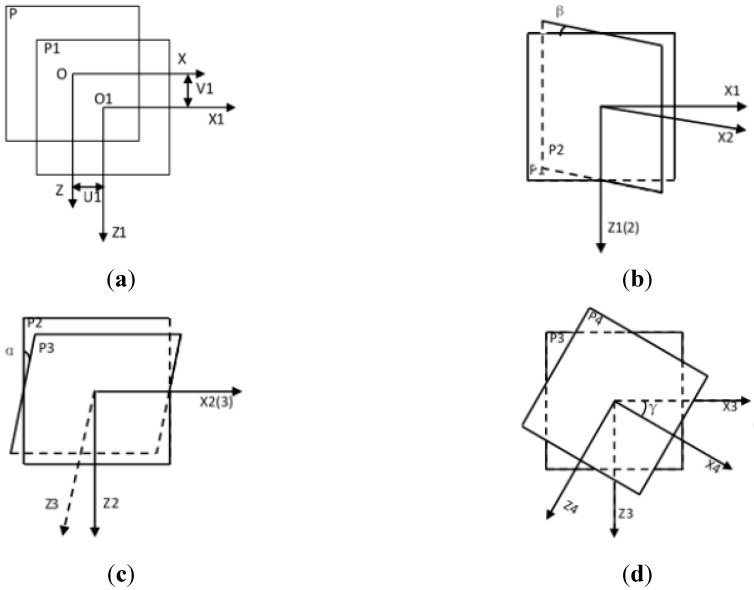
Misalignments of the detector. (**a**) The skewing of detector along X and Z axis; (**b**) the angle of detector tilt around Z axis; (**c**) the angle of detector tilt around X axis; and (**d**) the angle of detector tilt around Y axis.

All the misalignments of the detector are shown in Figure 2.

## 3. Principles

**Figure 3:**
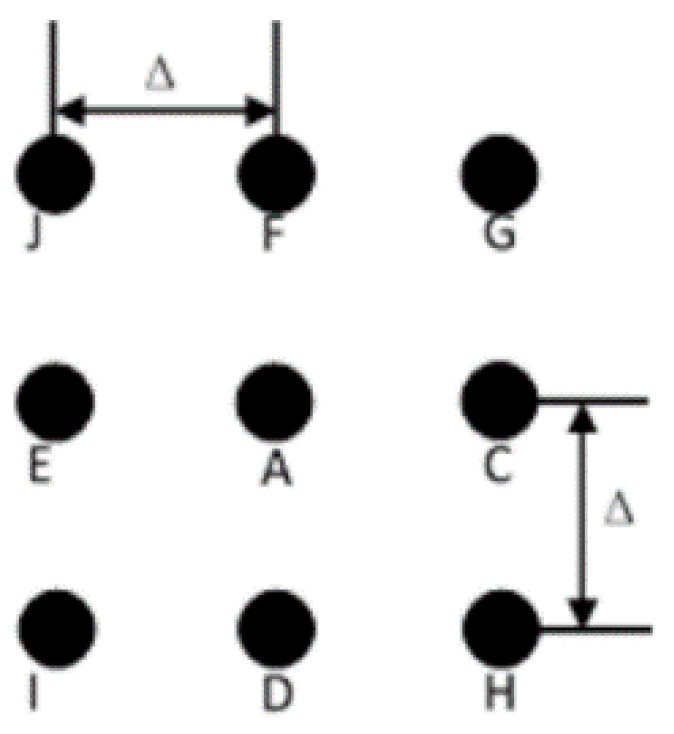
One plane of the phantom.

To estimate all the parameters, the calibration is carried out in several steps. Firstly, we place the phantom in the middle of the rotation table and take a projection of the phantom. One plane of the phantom is shown as Figure 3, it consists of nine ruby balls which are fixed on a carbon plate. Among these balls, G, H, I, and J are placed at the four vertexes of a square; C, D, E, and F are placed at the middle of the four edges; A is located at the center of the square; and the distance between two neighboring balls at the horizontal or vertical direction equals to Δ. The X-ray beam passes through the phantom and the nine balls are projected onto the detector. In the ideal case, the nine projection circles should be still on the four vertexes, the four midpoints and the center. However, when the system is not under ideal conditions, the positional relations of the projection circles will be changed, and we can solve the geometry parameters from the positions of the projection circles.

Secondly, extract the centers of the projection circles, and calculate the distances between every two circles. Learning from [8], we know that the distances of IJ and HG is decided by the angle of β. By calculating the ratio of the distances of AC, AE and AF, AD, we can establish the relationships between α and β. According to the coordinates of the D and F, γ can be obtained.

Thirdly, the values of SOD and SDD can be gotten by using the distances of DF and D1F1. Subsequently, the other four parameters can be solved according to the coordinates of the A0 and A10.

Lastly, adjust the system according to the parameters solved. Then do all the steps again until the parameters approaching to zero or meeting demands.

The details will be described in the following parts.

### 3.1. Calculation of α, β, γ

To describe the misalignments of the scanner, four right-handed systems of Cartesian coordinates are defined, referring to Appendix A. According to the coordinate systems established, the coordinates of all the ruby balls in each coordinate system are obtained. In addition, the coordinates of all the centers of the projection circles can be written out, seeing Appendix B.

According to coordinates of the centers of the projection circles and Euclidean distance between two points, the relationship of the distances is given by:

(1) $$\frac{\left|A_{0}C_{0}\right|}{\left|A_{0}E_{0}\right|}=\frac{Y_{S}-Y_{A}-\cos\text{α}\cdot \sin\text{β}\cdot \Delta }{Y_{S}-Y_{A}+\cos\text{α}\cdot \sin\text{β}\cdot \Delta }=a$$

(2) $$\frac{\left|A_{0}D_{0}\right|}{\left|A_{0}F_{0}\right|}=\frac{Y_{S}-Y_{A}+\sin\text{α}\cdot \Delta }{Y_{S}-Y_{A}-\sin\text{α}\cdot \Delta }=b$$

and hence:

(3) $$\sin\text{β}=\frac{1-a}{a+1}\cdot \frac{b+1}{b-1}\cdot \tan\text{α}$$

Sun *et al*. [8] proposed the formula:

(4) $$\frac{EF}{E^{\prime }F^{\prime }}=\frac{2\cdot f-l\cdot \tan\varphi }{2\cdot f+l\cdot \tan\varphi }$$

and it is suitable for TPB phantom. Apply Equation (4) to TPB phantom, and the ratio is written as;

(5) $$\frac{\left|IJ\right|}{\left|HG\right|}=\frac{2\cdot f+l\cdot \tan\text{β}}{2\cdot f-l\cdot \tan\text{β}}=c$$

where:

(6) l=2⋅Δ

Then we have:

(7) $$\tan\text{β}=\frac{f\cdot (c-1)}{\Delta \cdot (c+1)}$$

The accurate value of SOD is unknown, therefore, its estimated value f is used instead.

Combined Equation (1) with Equation (2), the tangent value of α becomes:

(8) $$\tan\text{α}=\sin(arc\tan\text{β})\cdot \frac{a+1}{1-a}\cdot \frac{b-1}{b+1}$$

According to Equation (2), we derive:

(9) $$Y_{S}-Y_{A}=\frac{b+1}{b-1}\cdot \sin\text{α}\cdot \Delta$$

Normally, with careful designing and installing, u2, v2, and β can be controlled, such as, u2 and v2 are smaller than 2 mm, and β is smaller than 2°. Utilize the coordinates of YS and YA in coordinate system P4 and see that:

(10) $$Y_{S}-Y_{A}=-u_{2}\cdot \cos\text{α}\cdot \sin\text{β}+f\cdot \cos\text{α}\cdot \cos\text{β}+v_{2}\cdot \sin\text{β}\approx f\cdot \cos\text{α}\cdot \cos\text{β}$$

As we can’t extract the projection point of the center of the balls without errors, when the angle of β is very small, the value of a (definition in Equation (1)) will be close to 1, so the Equation (8) will have a large error because of the inaccurate of a. On this occasion (when β is very small, such as β < 0.1°), the following equation is assumed:

(11) f⋅cosα⋅cosβ≈f⋅cosα

Then:

(12) $$\frac{b+1}{b-1}\cdot \sin\text{α}\cdot \Delta \approx f\cdot \cos\text{α}\cdot \cos\text{β}\approx f\cdot \cos\text{α}$$

(13) $$\tan\text{α}=\frac{(b-1)\cdot f}{(b+1)\cdot \Delta }$$

As the points D and F represent the axis of the rotation, the tangent value of γ can be written as:

(14) $$\tan\gamma =\frac{X_{F_{0}}-X_{D_{0}}}{Z_{D_{0}}-Z_{F_{0}}}$$

### 3.2. Calculation of u1, v1, u2, v2, SOD, SDD

In the coordinate system P4, the coordinate of projection of point A can be gotten by using the following equations:

(15) $${\text{X}}_{A_{10}}=\frac{Y_{S}\cdot X_{A_{1}}-Y_{A_{1}}\cdot X_{S}}{Y_{S}-Y_{A_{1}}}=\frac{Y_{S}\cdot (X_{A}-\sin\text{β}\cdot \cos\gamma \cdot \Delta -\sin\text{α}\cdot \cos\text{β}\cdot \sin\gamma \cdot \Delta )-X_{S}(Y_{A}-\cos\text{α}\cdot \cos\text{β}\cdot \Delta )}{Y_{S}-Y_{A}+\cos\text{α}\cdot \cos\text{β}\cdot \Delta }$$

(16) $${\text{Z}}_{A_{10}}=\frac{Y_{S}\cdot Z_{A_{1}}-Y_{A_{1}}\cdot Z_{S}}{Y_{S}-Y_{A_{1}}}=\frac{Y_{S}\cdot (Z_{A}-\sin\text{β}\cdot \sin\gamma \cdot \Delta +\sin\text{α}\cdot \cos\text{β}\cdot \cos\gamma \cdot \Delta )-Z_{S}(Y_{A}-\cos\text{α}\cdot \cos\text{β}\cdot \Delta )}{Y_{S}-Y_{A}+\cos\text{α}\cdot \cos\text{β}\cdot \Delta }$$

Equations (15) and (16) can be rewritten in the form:

(17) $$\begin{array}{l} Y_{S}\cdot (\sin\text{β}\cdot \cos\gamma \cdot \Delta +\sin\text{α}\cdot \cos\text{β}\cdot \sin\gamma \cdot \Delta )-X_{S}\cdot \cos\text{α}\cdot \cos\text{β}\cdot \Delta \\ =(Y_{S}-Y_{A})\cdot (X_{A_{0}}-X_{A_{10}})-\cos\text{α}\cdot \cos\text{β}\cdot \Delta \cdot X_{A_{10}} \end{array}$$

(18) $$\begin{array}{l} Y_{S}\cdot (-\sin\text{β}\cdot \sin\gamma \cdot \Delta +\sin\text{α}\cdot \cos\text{β}\cdot \cos\gamma \cdot \Delta )-Z_{S}\cdot \cos\text{α}\cdot \cos\text{β}\cdot \Delta \\ =(Y_{S}-Y_{A})({\text{Z}}_{A_{10}}-{\text{Z}}_{A_{0}})+\cos\text{α}\cdot \cos\text{β}\cdot \Delta \cdot {\text{Z}}_{A_{10}} \end{array}$$

Combined with Equation (10), the values of Δμ (which equals to u2 minus u1) and Δv (which is equal to v2 minus v1) could be solved.

As the distance between two points is not affected by the angle of γ, the angle of γ is assumed to be zero. From the Euclidean distance between two points, the distance between point D and point F can be described as:

(19) $$\begin{array}{l} DF^{2}=\frac{4\cdot {\left(Y_{S}-Y_{A}\right)}^{2}\cdot \Delta^{2}}{\left[{\left(Y_{S}-Y_{A}\right)}^{2}-{\left(\sin\text{α}\cdot \Delta \right)}^{2}\right]}\cdot \{{\left[X_{A_{0}}\cdot \sin\text{α}-(u_{2}-u_{1})\cdot \sin\text{α}\cdot \cos\text{β}-SDD\cdot \sin\text{α}\cdot \sin\text{β}\right]}^{2}\\ +\;{\left[Z_{A_{0}}\cdot \sin\text{α}+(u_{2}-u_{1})\cdot \sin\text{β}-SDD\cdot \cos\text{β}\right]}^{2}\} \end{array}$$

Substituting the value of DF to Equation (19), the value of SDD can be computed.

The distance of D1F1 is given as follows by using the same method:

(20) $$\begin{array}{l} D_{1}{F_{1}}^{2}=\frac{4\cdot {\left(Y_{S}-Y_{A_{1}}\right)}^{2}\cdot \Delta^{2}}{\left[{\left(Y_{S}-Y_{A_{1}}\right)}^{2}-{\left(\sin\text{α}\cdot \Delta \right)}^{2}\right]}\cdot \{{\left[X_{A_{10}}\cdot \sin\text{α}-(u_{2}-u_{1})\cdot \sin\text{α}\cdot \cos\text{β}-SDD\cdot \sin\text{α}\cdot \sin\text{β}\right]}^{2}\\ +{\left[Z_{A_{10}}\cdot \sin\text{α}+(u_{2}-u_{1})\cdot \sin\text{β}-SDD\cdot \cos\text{β}\right]}^{2}\} \end{array}$$

As the uncertainty of the parameters is influenced by the accurate of extracting of centers of balls which can’t be extracted without errors, we assume that Equation (21) is right when the angles of β and α is small (such as α and β are all smaller than 0.1°):

(21) $$Y_{S}-Y_{A}\;\approx f\cdot \cos\text{α}\cdot \cos\text{β}=\text{SOD}\cdot \cos\text{α}\cdot \cos\text{β}$$

Substituting Equation (21) to Equations (19) and (20), the accurate values of SOD and SDD can be gotten.

According to the coordinate of A0, we have:

(22) $$\begin{array}{l} {}[\sin\text{α}\cdot \sin\text{β}\cdot \sin\gamma \cdot Y_{S}-\cos\text{β}\cdot \cos\gamma \cdot Y_{S}-\cos\text{α}\cdot \sin\text{β}\cdot X_{S}]\cdot \mu_{1}+[\cos\text{α}\cdot \sin\gamma \cdot Y_{S}+\sin\text{α}\cdot X_{S}]\cdot \upsilon_{1}\\ =(Y_{S}-Y_{A})\cdot X_{A_{0}}-(SDD-SOD)\cdot [\sin\text{β}\cdot \cos\gamma \cdot Y_{S}+\sin\text{α}\cdot \cos\text{β}\cdot \sin\gamma \cdot Y_{S}-\cos\cdot \text{α}\cos\text{β}\cdot X_{S}] \end{array}$$

(23) $$\begin{array}{l} {}[-\sin\text{α}\cdot \sin\text{β}\cdot \cos\gamma \cdot Y_{S}-\cos\text{β}\cdot \sin\gamma \cdot Y_{S}-\cos\text{α}\cdot \sin\text{β}\cdot Z_{S}]\cdot \mu_{1}+[-\cos\text{α}\cdot \cos\gamma \cdot Y_{S}+\sin\text{α}\cdot Z_{S}]\cdot \upsilon_{1}\\ =(Y_{S}-Y_{A})\cdot Z_{A_{0}}-(SDD-SOD)\cdot [\sin\text{β}\cdot \sin\gamma \cdot Y_{S}-\sin\text{α}\cdot \cos\text{β}\cdot \cos\gamma \cdot Y_{S}-\cos\cdot \text{α}\cos\text{β}\cdot Z_{S}] \end{array}$$

Then we can solve the values of u1 and v1. By using the values of Δμ and Δv, the values of u2 and v2 also could be computed.

So far, all the parameters we need are solved by using the coordinates of the corresponding projections.

## 4. Simulations

**Figure 4:**
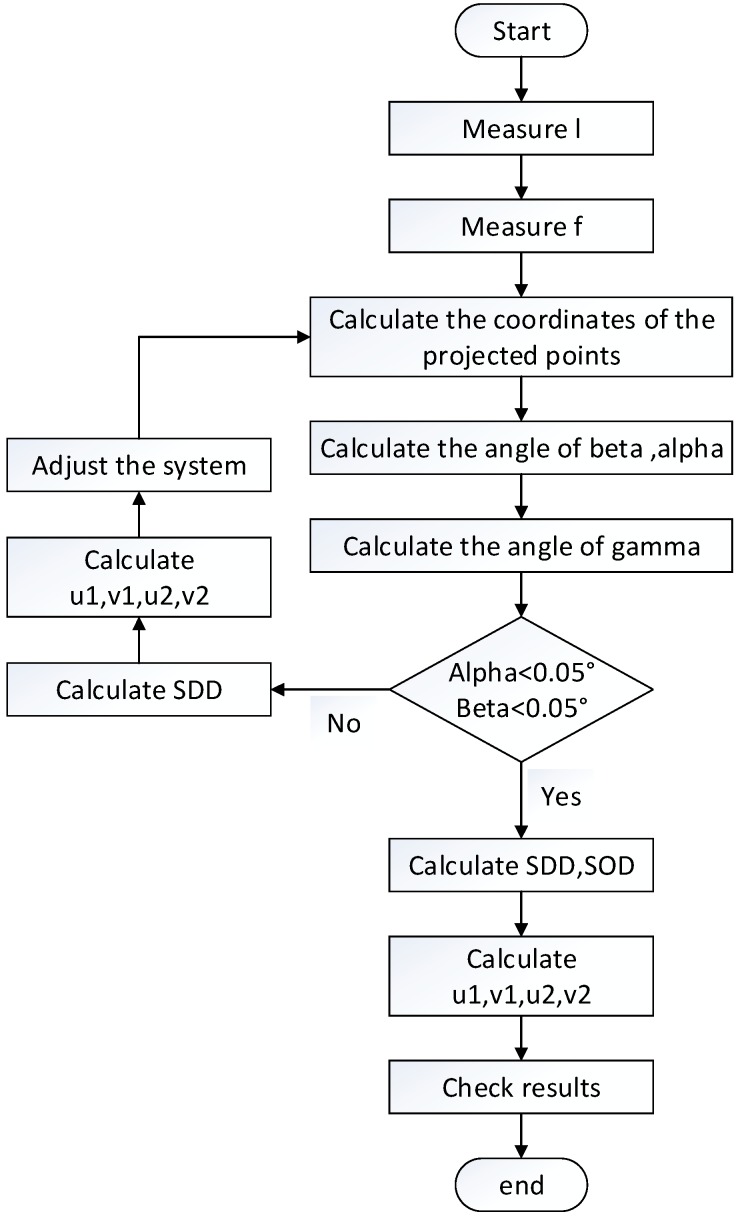
The procedure of calibration.

The whole procedure of calibration is shown as Figure 4. All of the symbols shown in the figure have been introduced in Section 2.

To validate the accuracy of the calibration method described in Section 3, the projection coordinates of the TPB on the detector were simulated. The calibration method was then applied on the simulated data and the calculated system parameters were compared with setting values. The main imaging parameters of typical scanner geometry in the simulation study are shown as Table 1.

We implement the simulation in two steps.

Firstly, we simulate the center coordinates of the projection circles by using MATLAB [15], and in this way, we can get the centers with no errors. We have simulated a misalignment of the X-ray source, and the detector when the coordinates of all the projected points are measured accurately and the results are listed in Table 2. In theory, there should be no errors when the centers of the balls are exactly given. However, there still exist small errors between the computed misalignments by our method and setting values, which is caused by ignoring the parameters of α and β when they were less than 0.1°. These caused the small errors between true values and simulating results.

**Table 1:** The main parameters of CT system.

SOD	30 mm
SDD	300 mm
Resolution of the detector	1024 × 1024
Pixel size	127 µm × 127 µm
Distance between neighboring balls in horizontal or vertical direction	4 mm
Diameter of the balls	3 mm

**Table 2:** Simulation results without errors in extracting centers.

Parameters	SOD (mm)	SDD (mm)	α (°)	β (°)	γ (°)	u_1_ (μm)	v_1_ (μm)	u_2_ (μm)	v_2_ (μm)
True values	30	300	2	1.5	1.2	100	60	160	210
simualting results	30	300	1.9979	1.4984	1.2001	101.18	61.66	159.87	209.82
Residual Errors	0	0	−0.0021	−0.0016	0.0001	1.18	1.66	−0.13	−0.18

However, it is impossible to measure the coordinates of all the projected points without errors. To analyze the influence of the accuracy of the centers on the results, we simulated the system with known errors secondly. We simulate the scanner system and acquire the projection of one plane of the phantom [16]. When the scanner has misalignments, according to the definition of conic section, the projection of a ball on the detector is an ellipse. When the misalignments are not very large, the center of the projected ellipse approaches the point of the projection of the center of the ball, though it is not equal. So we use the center of the projected ellipses to replace the projection of the centers of the balls in the phantom. The result is listed in Table 3.

**Table 3:** Simulation results with errors in extracting centers.

Parameters	SOD (mm)	SDD (mm)	α (°)	β (°)	γ (°)	u_1_ (μm)	v_1_ (μm)	u_2_ (μm)	v_2_ (μm)
Given	30	300	2	1.5	1.2	100	60	160	210
Solved	29.847	299.081	2.0085	1.4854	1.2109	99.37	72.98	153.15	207.82
Residual Error	−0.153	−0.919	0.0085	−0.0146	0.0109	−0.63	12.98	−6.85	−3.18

Seen from Table 3, the errors of SOD, SDD, α, β, and γ are less than the other four. The result of the phenomenon is that a small error of the coordinates of the balls will result in little errors of the length between two points. Since the five parameters (SOD, SDD, α, β, γ) are the functions of lengths between the points, so they are less influenced by the errors of the coordinates of the balls. On the other hand, the other four (u1, v1, u2, v2) are functions of the coordinate of the projection of point A, so they are affected by the errors more obviously. However, through this method we can adjust the system to a relatively ideal condition which can satisfy the requirement of the method.

## 5. Experiments

To verify the effect of the calibration method in realistic imaging system, an experiment was performed in our micro-CT system with unknown misalignments. After adjusting the system under the guidance of geometric calibration using the proposed method in this article, a sample was scanned. The tomographic volume data are reconstructed using the FDK algorithm [17].

The micro-CT system in our laboratory consists of a micro-focus X-ray source (XWT-160-TC, Worx, transmission, Germany), a high-precision rotation stage and a Flat Panel Detector (1313DX, Varian Medical, Systems, Palo Alto, CA, USA), all of which are mounted on an optical bench. The size of the focal spot of the X-ray source is about 1.5 µm and the best resolution is 0.3 µm. The image detector array has an active area of 13.0 × 13.0 cm with a 1024 × 1024 matrix and a pixel size of 127 μm. The estimated values of SOD and SDD are set to 10 mm and 100 mm in the data acquisition software roughly. The phantom we used is manufactured by National Institute of Metrology, China. The accurate distances between the balls on the phantom are measured by using micro-nano coordinate measuring machine (F25, Carl Zeiss, Germany).

**Figure 5:**
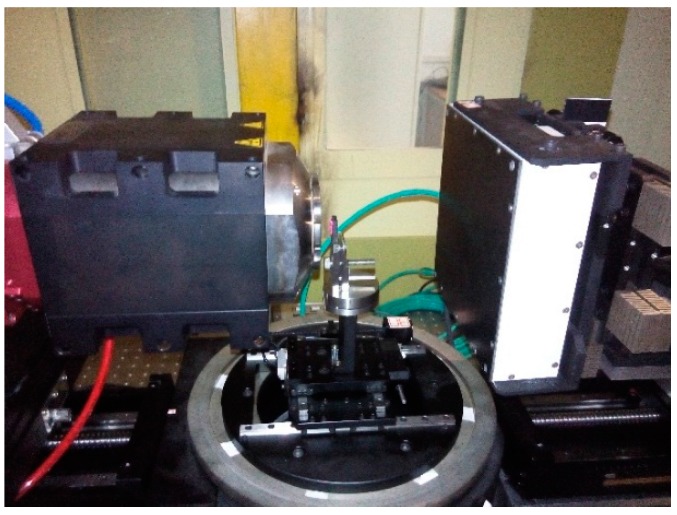
Photograph of the micro-CT system with a micro-focus X-ray source, rotation stage, and a flat panel detector.

**Figure 6:**
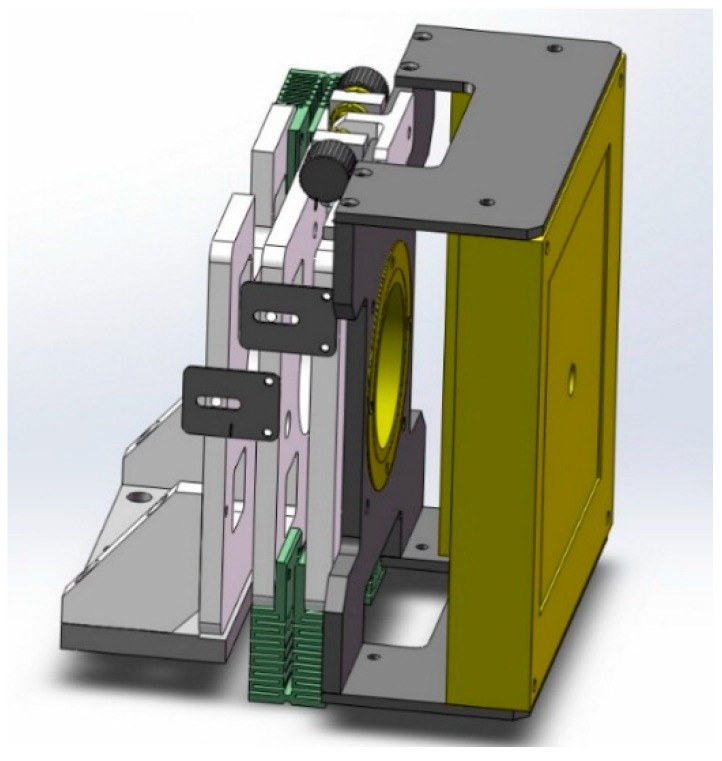
Design of the adjusting mechanism to adjust the location of the detector.

All the steps listed in Figure 4 were implemented in our Micro-CT system. Figure 5 is a photograph of the micro-CT system we used and the set of geometric parameters is solved by use of the presented method. We adjusted the position of the detector every time after the calibration done by using the equipment shown as Figure 6. It can adjust the three angles of the detector. In our micro-CT, the horizontal and vertical direction of the X-ray source and detector also can be adjusted by precision displacement units.

**Figure 7:**
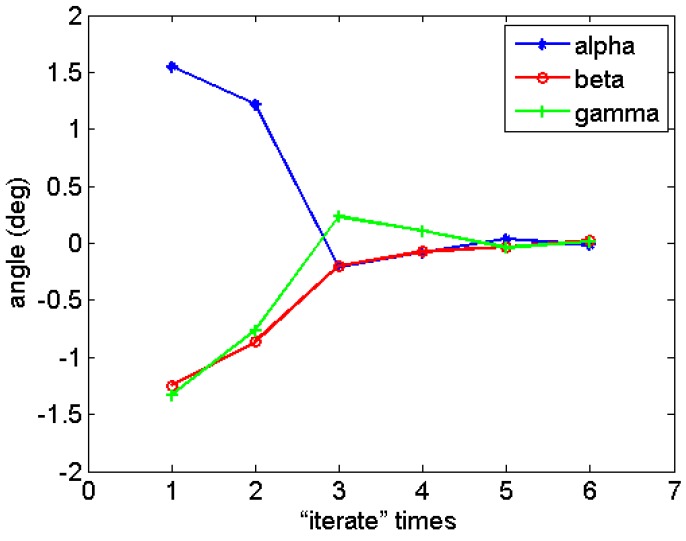
The values of the three angles varying with the “iterate” times.

The calibration results are shown in Table 4. The curves description the values of α, β, and γ are shown in Figure 7. As seen from this, the values of the three angles decrease quickly, and the minima is less than 0.1°. The results indicate that the residuals of geometric parameters become small and our method is reliable.

**Table 4:** Calibration results of all the parameters.

	α (°)	β (°)	γ (°)	SOD (mm)	SDD (mm)	u_1_ (μm)	v_1_ (μm)	u_2_ (μm)	v_2_ (μm)
1#	1.5516	−1.2452	−1.3332						
2#	1.2185	−0.863	−0.7666						
3#	−0.201	−0.1967	0.2328						
4#	−0.0824	−0.0764	0.1084	11.458	116.1	−580	9577	135	55
5#	0.0357	−0.0351	−0.0361	11.445	115.7	−512	9460	134	52

**Figure 8:**
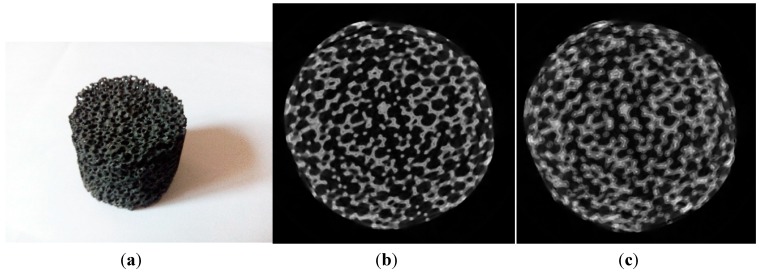
Scanned sample and the middle slice of the volume data. (**a**) SiC sample; (**b**) the middle slice after system adjustment; (**c**) the middle slice before system adjustment.

After we adjusted the system using experimental methods, and before the method proposed here, a SiC sample was scanned and reconstructed through using the estimated parameters. After we adjusted the system by using the geometric parameters as guidance, it was scanned and reconstructed again. Figure 8 displays micro-CT images of the same slice of the SiC sample before and after adjusting. It can be obviously observed that the image quality after system adjustment is much higher than before, which demonstrates that the calibrated method proposed in this paper is efficient.

## 6. Conclusions

This paper addresses a new “iterative” method to estimate geometric parameters of micro-CT. The method can estimate all the parameters by acquiring only one projection of the designed PTB phantom. Compared with other methods, it doesn’t need to turn the rotation stage and avoids the radial or axial errors of the rotation stage. In addition, there is no restriction on any one of the parameters. It only needs 3–5 “iterate” times to adjust the system to almost ideal condition, and the three angle parameters decrease quickly. The accuracy of this method is confirmed by simulated and experimental results. The simulated results show that there are only small errors with accurately extracting the centers of the projected balls and it is efficient. At last, a SiC sample was scanned and reconstructed. The experiments results show that the artifacts are reduced and image quality is improved. Future research will concentrate on studying methods to eliminate the influence of focal spot drifts.

## Appendix A

Similar to the coordinate system X, Y and Z, to describe the misalignments of the scanner, four right-handed systems of Cartesian coordinates are defined. The first one is X1, Y1, and Z1 attached to the plane P1, which denotes the ideal coordinate P translating u1, v1 along X and Z. The second one is X2, Y2 and Z2 attached to the plane P2, which denotes the coordinate P1 twisting angle beta along Z1. The third one is X3, Y3 and Z3 attached to the plane P3, which denotes the coordinate P2 twisting angle alpha along X2. And the last one is X4, Y4 and Z4 attached to the plane P4, which denotes the coordinate P3 twisting angle gamma along Y3.

According to the coordinate systems established above, the relationship of all the coordinate systems is built. We define the translation matrix from P to P1 as T1, from P1 to P2 as T2, from P2 to P3 as T3, from P3 to P4 as T4, then we can get the translation matrixes which is formulated by α, β, γ, u1 and v1.

(A1) $$P_{3}=(P+T_{1})•T_{2}•T_{3}$$

(A2) $$P_{4}=(P+T_{1})•T_{2}•T_{3}•T_{4}$$

(A3) $$T_{1}=\left(-\mu_{1},0,-\upsilon_{1}\right)$$

(A4) $$T_{2}=\left(\begin{array}{ccc} \cos\text{β} & -\sin\text{β} & 0\\ \sin\text{β} & \cos\text{β} & 0\\ 0 & 0 & 1 \end{array}\right)$$

(A5) $$T_{3}=\left(\begin{array}{ccc} 1 & 0 & 0\\ 0 & \cos\text{α} & -\sin\text{α}\\ 0 & \sin\text{α} & \cos\text{α} \end{array}\right)$$

(A6) $$T_{4}=\left(\begin{array}{ccc} \cos\gamma & 0 & \sin\gamma \\ 0 & 1 & 0\\ -\sin\gamma & 0 & \cos\gamma \end{array}\right)$$

From above analysis, the coordinate of source in the coordinate system P can be written as S (u2, SDD, v2). The distance between the first plane of the phantom and the plane XOZ is ODD, which equals to SDD minus SOD. Then all the coordinates of the 18 balls in the coordinate system P can be expressed as:

(A7) $$\begin{array}{ll} A:(0,d-f,0) & A_{1}:(0,d-f-\Delta ,0)\\ C:(\Delta ,d-f,0) & C_{1}:(\Delta ,d-f-\Delta ,0)\\ D:(0,d-f,\Delta ) & D_{1}:(0,d-f-\Delta ,\Delta )\\ E:(-\Delta ,d-f,0) & E_{1}:(-\Delta ,d-f-\Delta ,0)\\ F:(0,d-f,-\Delta ) & F_{1}:(0,d-f-\Delta ,-\Delta )\\ G:(\Delta ,d-f,-\Delta ) & G_{1}:(\Delta ,d-f-\Delta ,-\Delta )\\ H:(\Delta ,d-f,\Delta ) & H_{1}:(\Delta ,d-f-\Delta ,\Delta )\\ I:(-\Delta ,d-f,\Delta ) & I_{1}:(-\Delta ,d-f-\Delta ,\Delta )\\ J:(-\Delta ,d-f,-\Delta ) & J_{1}:(-\Delta ,d-f-\Delta ,-\Delta ) \end{array}$$

According to Equations (1) and (2), the coordinates of all the points in coordinate system P3 can be written out. Taking the point A as an example, its coordinate in coordinate system P3 can be expressed as:

(A8) $$A={\left(\begin{array}{l} -\cos\text{β}\cdot \mu_{1}+\sin\text{β}\cdot (d-f)\\ \cos\text{α}\cdot \sin\text{β}\cdot \mu_{1}+\cos\text{α}\cdot \cos\text{β}\cdot (d-f)-\sin\text{α}\cdot \upsilon_{1}\\ -\sin\text{α}\cdot \sin\text{β}\cdot \mu_{1}-\sin\text{α}\cdot \cos\text{β}\cdot (d-f)-\cos\text{α}\cdot \upsilon_{1} \end{array}\right)}^{T}={\left(\begin{array}{l} X_{A}\\ Y_{A}\\ Z_{A} \end{array}\right)}^{T}$$

## Appendix B

As the angle γ do not change the distances between every two projection circle centers, here we assume γ is zero. We define the line passing through point S and point A intersects with the detector at point A0, and the same definition to points C0~J0.

In the coordinate system P3, we have

(B1) $$Y_{A_{0}}=\cdots =Y_{J_{0}}=0$$

Taking point A as an example, seeking for the coordinate of A0.

(B2) $$\frac{Y_{S}-Y_{A}}{Y_{A}-Y_{A_{0}}}=\frac{X_{S}-X_{A}}{X_{A}-X_{A_{0}}}=\frac{Z_{S}-Z_{A}}{Z_{A}-Z_{A_{0}}}$$

From formulas above, we can get

(B3) $$X_{A_{0}}=\frac{Y_{S}\cdot X_{A}-Y_{A}\cdot X_{S}}{Y_{S}-Y_{A}}$$

(B4) $$Z_{A_{0}}=\frac{Y_{S}\cdot Z_{A}-Y_{A}\cdot Z_{S}}{Y_{S}-Y_{A}}$$

## Appendix C

The same to point A0, we can acquire all the coordinates of points C0~J0. Using these coordinates, the distances between every two points can be gotten. So we have

(C1) $$\frac{{\left|A_{0}C_{0}\right|}^{2}}{{\left|A_{0}E_{0}\right|}^{2}}=\frac{{\left(Y_{S}-Y_{E}\right)}^{2}}{{\left(Y_{S}-Y_{C}\right)}^{2}}=\frac{{\left(Y_{S}-Y_{A}-\cos\text{α}\cdot \sin\text{β}\cdot \Delta \right)}^{2}}{{\left(Y_{S}-Y_{A}+\cos\text{α}\cdot \sin\text{β}\cdot \Delta \right)}^{2}}$$

(C2) $$\frac{{\left|A_{0}D_{0}\right|}^{2}}{{\left|A_{0}F_{0}\right|}^{2}}=\frac{{\left(Y_{S}-Y_{F}\right)}^{2}}{{\left(Y_{S}-Y_{D}\right)}^{2}}=\frac{{\left(Y_{S}-Y_{A}+\sin\text{α}\cdot \Delta \right)}^{2}}{{\left(Y_{S}-Y_{A}-\sin\text{α}\cdot \Delta \right)}^{2}}$$

In general, the angles of twisting will not be too large, so we can affirm that

(C3) $$Y_{S}-Y_{A}>0$$

Then, the above formulas can translate to

(C4) $$\frac{\left|A_{0}C_{0}\right|}{\left|A_{0}E_{0}\right|}=\frac{Y_{S}-Y_{A}-\cos\text{α}\cdot \sin\text{β}\cdot \Delta }{Y_{S}-Y_{A}+\cos\text{α}\cdot \sin\text{β}\cdot \Delta }=a$$

(C5) $$\frac{\left|A_{0}D_{0}\right|}{\left|A_{0}F_{0}\right|}=\frac{Y_{S}-Y_{A}+\sin\text{α}\cdot \Delta }{Y_{S}-Y_{A}-\sin\text{α}\cdot \Delta }=b$$
